# Thyroid Oncology

**DOI:** 10.4061/2011/467570

**Published:** 2011-10-09

**Authors:** Maria João M. Bugalho, Nelson Wohllk, Ana O. Hoff, Maria E. Cabanillas

**Affiliations:** ^1^Serviço de Endocrinologia, Instituto Português de Oncologia, 1099-023 Lisboa, Portugal; ^2^Faculdade de Ciências Médicas, Universidade Nova de Lisboa, 1169-056 Lisboa, Portugal; ^3^Seccion Endocrinologia, Hospital del Salvador, Santiago de Chile, Universidad de Chile, Chile; ^4^Departamento de Endocrinologia, Instituto do Cancer do Estado de São Paulo, Faculdade de Medicina da Universidade de São paulo, São Paulo, Brazil; ^5^Department of Endocrine Neoplasia and Hormonal Disorders, University of Texas M.D. Anderson Cancer Center, Houston, TX 77030, USA

We are pleased to bring you the Special Issue of the Journal Thyroid Research dedicated to Thyroid Oncology.

The incidence of thyroid cancer has been increasing in recent decades mainly due to an increase in papillary thyroid carcinomas (PTCs). Among these, tumors ≤1 cm increased the most. Whether this represents a higher sensitivity to detect smaller tumors or depends on other factors such as environmental factors remains unclear [1–3]. 

According to the World Health Organization (WHO), papillary thyroid carcinomas measuring 1 cm or less are designated as papillary thyroid microcarcinomas (PTMCs).

Incidentally diagnosed PTMCs are generally indolent tumors. However, PTMCs detected due to clinically suspected and histological confirmed lymph node metastases or associated with extra thyroidal extension may have a more aggressive behavior [4, 5]. Thus, it is inaccurate and misleading to regard all PTMCs patients as having the same level of risk. Most studies based their conclusions on clinicopathological factors. Recently, Kim et al. [6] showed that the gene expression profiles of PTMCs were not different from those of larger PTCs and suggested that PTMCs may represent an earlier stage of the same disease. 

Differences in the form of presentation between papillary microcarcinomas and papillary carcinomas of larger size are discussed in this issue by C. Zafon et al., who concluded that patients with a low aggressive profile were significantly older than the remaining patients. This interesting finding awaits confirmation by other studies and larger series. 

Fine-needle aspiration cytology (FNAC) of thyroid nodules is highly sensitive in the diagnosis of papillary, medullary, and anaplastic carcinomas. Distinction between benign lesions, such as follicular adenoma or nodular adenomatous goiter, and follicular carcinoma or follicular variant of papillary carcinoma remains a problem. The final diagnosis depends on histological evaluation. 

The study by M. Bonzanini et al. addresses practical issues related to the existence of different FNAC classifications [7, 8] and was designed to retrospectively analyze the benefits of subclassifying the “undetermined” cytologic reports into two categories: “follicular lesion” (FL) and “atypia of undetermined significance” (AUS). Data obtained on this basis indicate that AUS is associated with higher malignancy rate than FL. Moreover, the authors provide data in favor of an integrated analysis of clinical, cytological, biochemical, and ecographic findings to improve diagnostic accuracy. 

Young patients with differentiated thyroid carcinoma (DTC) represent a particular group. Childhood DTC is more frequently multicentric and is associated with a more locally aggressive and more frequent distant disease than its adult counterpart. Nonetheless, recent series [9], with long followup, have shown that fewer than 2% of children die from DTC contrasting to a much higher number of patients dying from nonthyroid malignancy. Further more, seventy-three percent of those who died from nonthyroid malignancy had received adjuvant radioactive iodine (^131^I) therapy. 

In children, the lungs are almost the sole distant metastatic site, and pulmonary metastases are nearly always functional [10]. 

A risk-stratified approach is probably the best choice to optimize treatment and reduce risks associated with therapy [11]. To choose among the classical risk stratification systems, the most adequate one for young patients is still a matter of debate. F. Vaisman et al. discuss these and other points. 

Medullary thyroid carcinoma (MTC) is a neuroendocrine tumor derived from parafollicular cells of the thyroid that occurs in both sporadic and hereditary forms. MTC spreads early to lymph nodes and is both chemo- and radioresistant. Early surgery is the only therapeutic approach potentially curative thus explaining the importance of an early detection.

Activating mutations of the rearranged during transfection (*RET*) proto-oncogene were first described in patients with familial forms in 1993 [12, 13]. Additionally, somatic *RET *mutations were identified in up to 65% of patients with sporadic MTC [14, 15]. The *RET* gene is located in chromosome 10q11.2 and codes for a tyrosine kinase (TK) receptor. These molecular advances made possible to define genotype-phenotype correlations; the International RET Mutation Consortium and the American Thyroid Association provided guidelines for the timing of prophylactic surgery based on genetic analysis [16, 17]. Moreover, promising targeted therapies have been developed for progressive and advanced MTC. 

Genetic screening became a routine, worldwide, in the management of MTC patients at a preclinical stage. Results presented by M. Hedayati et al. in the current issue, in addition to those previously presented by Alvandi et al. [18], are illustrative of the mutational profile observed among Iranian patients with MTC.

Based on the understanding of the altered molecular pathways underlying MTC, a number of “targeted” therapies have been developed. K. Gómez et al. present a comprehensive review of the most promising TK inhibitors for the treatment of MTC and draw attention to possible adverse effects and drug resistance.

Standard treatment of DTC includes surgery, ^131^I and thyroid hormone suppressive therapy. ^131^I, selectively targeting thyroid cells, was probably the first targeted therapy for cancer. For those cases refractory to^131^I and for patients with local aggressive or metastatic disease, until recently, there were no effective treatments. 

During the last decades, a large body of information has been generated on the molecular alterations, particularly on the role of oncogenic kinases involved in thyroid carcinomas. Based on this information, thyroid became, once more, a model for the use of new targeted therapies specially the kinase inhibitors. Interest in this field grew, and future holds promise. The role for combinatory treatments is still not defined.

Papers by H. Prazeres et al. and S. B. Bales et al. review genomic changes in thyroid cancer (DTC and MTC) and discuss how this information might be used to improve targeted therapies.

Epigenetic mechanisms are likely to play an important role in thyroid cancers particularly by modulating tumor progression. Whereas mutations generally alter intracellular signaling pathways, the epigenetic mechanisms may interfere with tumor environment as recently shown [19]. In this issue, O. P. Eze et al. present a thorough revision of this theme and discuss implications for future therapies designed to attain different pathways. 

Clinical trials of TK inhibitors in patients with advanced thyroid cancer have shown promising preliminary results, justifying enthusiasm among physicians and expectation among patients. The Food and Drug Administration recently approved Vandetanib for local advanced or metastatic MTC.

Despite the promising results, TK inhibitors have a broad spectrum of adverse effects. Considering that this class of therapeutic agents is to be used as chronic treatment, clinicians responsible for their use need to be familiar with adverse effects associated with TK inhibitors and prepared to manage them. M. E. Cabanillas et al. provide us with a comprehensive revision and practical tips to optimize treatment and minimize toxicity. 

We are grateful to all contributors, reviewers, and the editorial staff.

*Maria João M. Bugalho* *Maria João M. Bugalho* *Nelson Wohllk* *Nelson Wohllk* *Ana O. Hoff* *Ana O. Hoff* *Maria E. Cabanillas* *Maria E. Cabanillas*
